# Disclosure of sexual orientation to health professionals in China: results from an online cross-sectional study

**DOI:** 10.7448/IAS.20.1.21416

**Published:** 2017-02-06

**Authors:** Weiming Tang, Jessica Mao, Songyuan Tang, Chuncheng Liu, Katie Mollan, Bolin Cao, Terrence Wong, Ye Zhang, Michael Hudgens, Yilu Qin, Larry Han, Baoli Ma, Bin Yang, Wei Ma, Chongyi Wei, Joseph D Tucker, SESH Study Group

**Affiliations:** ^a^ University of North Carolina at Chapel Hill Project-China, Guangzhou, China; ^b^ SESH Study Group of University of North Carolina at Chapel Hill, Guangzhou, China; ^c^ Department of STI Control, Guangdong Center for Skin Diseases and STI Control, Guangzhou, China; ^d^ Department of Infectious Diseases, School of Medicine of University of North Carolina at Chapel Hill, Chapel Hill, NC, USA; ^e^ Department of Biostatistics of University of North Carolina at Chapel Hill, Chapel Hill, NC, USA; ^f^ Danlan Gongyi, Beijing, China; ^g^ School of Public Health, Shandong University, Jinan, China; ^h^ School of Medicine, University of California, San Francisco, San Francisco, CA, USA

**Keywords:** China, disclosure, healthcare professional, men who have sex with men (MSM), sexual behaviours, sexual orientation

## Abstract

**Background**: Many men who have sex with men (MSM) in China are “in the closet.” The low rate of disclosure may impact sexual behaviours, testing for HIV and other sexually transmitted infections (STIs), and diseases transmission. This study examines factors associated with overall sexual orientation disclosure and disclosure to healthcare professionals.

**Methods**: A nationwide cross-sectional online survey was conducted from September 2014 to October 2014 in China. Participants completed questions covering socio-demographic information, sexual behaviours, HIV/STI testing history, and self-reported HIV status. We defined healthcare professional disclosure as disclosing to a doctor or other medical provider.

**Results**: A total of 1819 men started the survey and 1424 (78.3%) completed it. Among the 1424 participants, 62.2% (886/1424) reported overall disclosure, and 16.3% (232/1424) disclosed to healthcare professionals. In multivariate analyses, the odds of sexual orientation disclosure were 56% higher among MSM who used smartphone-based, sex-seeking applications [adjusted odds ratio (aOR) = 1.56, 95% CI: 1.25–2.95], but were lower among MSM reporting sex while drunk or recreational drug use. The odds of disclosure to a healthcare professional were greater among MSM who had ever tested for HIV or STIs (aOR = 3.36, 95% CI: 2.50–4.51 for HIV, and aOR = 4.92, 95% CI: 3.47–6.96 for STIs, respectively) or self-reported as living with HIV (aOR = 1.59, 95% CI: 0.93–2.72).

**Conclusion**: Over 80% of MSM had not disclosed their sexual orientation to health professionals. This low level of disclosure likely represents a major obstacle to serving the unique needs of MSM in clinical settings. Further research and interventions to facilitate MSM sexual orientation disclosure, especially to health professionals, are urgently needed.

## Introduction

Many men who have sex with men (MSM) in low- and middle-income countries (LMICs) are “in the closet,” or have not disclosed their sexual orientation [1,2]. We define sexual orientation disclosure as having ever disclosed one’s sexual orientation to anyone other than a sexual partner, and healthcare professional disclosure as disclosing to a doctor or other medical provider. The large closeted MSM population in LMICs is likely related to social and cultural pressures of the local environment. MSM in these countries are subject to prejudice, stigma, and social discrimination by their communities [3]. As a result of these sociocultural pressures, MSM may face informal social condemnation, loss of employment, and loss of social standing [4,5]. In addition, the benefits of sexual orientation disclosure may be limited. Among men who disclose their sexual orientation, social support and MSM-specific clinical services are difficult to identify [6]. Nonetheless, sexual orientation disclosure among MSM has been associated with lower rates of depression [7], good peer support, and improved access to prevention services [8].

Much of the literature on MSM disclosure has come from high-income settings where local environments are often less harsh towards homosexuality [9]. The focus of these studies is usually to emphasize the potential benefits of disclosing sexual orientation to healthcare providers and to focus on ways to promote disclosure, social support, and reduction of stigma and discrimination [6,10,11]. In LMICs, there is less research on MSM sexual orientation disclosure [12]. The limited existing research has focused on MSM who are seen in clinics, overlooking a large number of men who do not disclose their sexual orientation to clinicians working in other settings or research staff.

Despite entrenched homophobia in many parts of China [13], there are slow cultural changes underway that create new opportunities for MSM sexual orientation disclosure. Over the last three decades, there have been progressive movements, liberalizing the country socially, culturally and economically, leading to more open and inclusive attitudes towards MSM [14]. As a result, there is growing recognition of the importance of clinical services tailored to meet the needs of MSM [15]. At the community level, there is an increasing number of community-based organizations (CBOs) focusing on promotion of sexual health among MSM [16] and MSM-friendly HIV testing programmes [17]. These developments may encourage Chinese MSM to disclose their sexual orientation. This study examined Chinese MSM sexual orientation disclosure and disclosure to healthcare professionals.

## Methods

### Study design and sampling methods

From September 2014 to October 2014, the University of North Carolina Project-China conducted a nationwide, online, cross-sectional study among Chinese MSM. Banner ads to the online survey were put on three gay websites targeting geographically disparate regions in China in order to recruit MSM from across the nation. Participants entered the survey by clicking on banner links. The three websites were Danlan Gongyi from Northern China (http://www.danlan.org), Yunnan Tongzhi from Southern China (http://www.yntz.net), and Jiangsu Tongzhi from Eastern China (http://www.jstz.org). We used a standardized checklist for reporting internet trial results [18].

Detailed study recruitment procedures have been reported elsewhere [19]. Participants who clicked the survey links on the websites were directed to the online survey that was hosted by Qualtrics (Provo, Utah). The first three questions of the survey screened for eligibility. Eligibility criteria included 16 years or older, born male, and ever having engaged in anal sex with a man. Eligible participants were then required to give informed consent prior to beginning the survey.

### Measures

The online survey was anonymous and collected information on socio-demographic information and sexual risk behaviours. Socio-demographic information included age (as a continuous variable and further categorized into three groups: less than 20, 20–29, or 30 and above), occupation (student or not), marital status (never married or ever married), education (high school or below, college/bachelors, or post-graduate), residence (urban or rural), and annual income (less than $3000 USD, $3001–6000 USD, $6001–10,000 USD, $10001–15,000 USD, or more than $15,000 USD). Participants were asked to report their self-identified sexual orientation (gay or bisexual) and their current self-identified gender (male or transgender). Participants were asked if they had ever tested for HIV (yes or no) and other STIs (yes or no) in their lifetime. Participants were asked whether or not they currently had a primary partner (yes or no), their preferred sexual role during anal sex (insertive, receptive, or no preference), if they had ever had vaginal or oral sex with women (yes or no), and whether they had condomless sex with a female partner in the last three months (yes or no). Other sexual risk behaviour questions included whether participants had participated in any condomless sex with men in the last 6 months (yes, no, or no anal sex in last 6 months), any sex while under the influence of alcohol in the last 3 months (yes or no), and any sex while using recreational drugs (including, but not limited to poppers or rush [amyl nitrite], ecstasy, crystal methamphetamine) in the last 12 months (yes or no). Finally, participants were also asked how they sought sex partners in the last 6 months, including smartphone-based sex-seeking apps (gay apps), other Internet resources, or in-person methods only.

With regards to sexual orientation disclosure, participants were asked whether they had disclosed their sexual orientation to anyone other than their partners (yes or no). If they answered yes, they were asked more questions about with whom they had disclosed their sexual orientation: friends, parents, other family members or relatives, co-workers, or doctors and other healthcare providers.

### Statistical analysis

For the descriptive analysis, we separated the socio-demographic and risk behaviour information by whether participants had disclosed their sexual orientation or not. Univariate and multivariate logistic regressions were used to evaluate factors associated with sexual orientation disclosure among the study participants. Factors that were adjusted in the multivariate analyses included age (continuous), residence (urban or rural), education level (high school or below, college, or post-graduate), and annual income (less than $3000 USD, $3001–6000 USD, $6001–10,000 USD, $10,001–15,000 USD, or more than $15,000 USD). Models were built using the results of a literature search and prior knowledge from previous work of the study group and collaborators to select potential confounders. A universal directed acyclic graph (DAG) was drawn using this data. Finally, based on this selection process, age, residence, educational level and annual income were included in the final models.

All data analyses were completed using SAS 9.4 (SAS int. Cary, NC, USA). We used similar methods to evaluate the factors correlated with sexual orientation disclosure to healthcare professionals.

### Ethical statement

Ethical approval was attained from the ethics review committees at the Guangdong Provincial Center for Skin Diseases and STI Control (Guangzhou, China), University of North Carolina at Chapel Hill (North Carolina, USA), and the University of California, San Francisco (California, USA) prior to the launch of the survey.

## Results

A total of 1819 men started the survey and a total of 1424 (78.3%) participants met the inclusion criteria completed the online survey.

### Socio-demographics and sex behaviours

The mean age of the 1424 participants was 25.6 ± 6.8 years old, the majority of whom (77.5%, *n* = 1104) were under 30 years old. Overall, 25.9% (*n* = 369) of men had completed less than high school and 16.2% (*n* = 230) of them were married. In addition, 43.7% (*n* = 511) of participants were currently enrolled as full-time or part-time students, and the majority of participants (81.9%, *n* = 1166) had an annual income of less than $10000 USD (Table 1).

**Table 1.** Demographic characteristics and behaviours among Chinese MSM, 2014 (*N* = 1424).

		Disclosers (*n* = 886)		Non-disclosers (*N* = 538)		Overall (*N* = 1424)	
		Frequency	Per cent (95% CI)	Frequency	Per cent (95% CI)	Frequency	Per cent
Age	*<20*	147	16.6(14.1,19.0)	59	11.0(8.3,13.6)	206	14.5
	*20–29*	561	63.3(60.1,66.5)	337	62.6(58.5,66.7)	898	63.1
	*≥30*	178	20.1(17.4,22.7)	142	26.4(22.7,30.1)	320	22.5
Marital status	*Never married*	776	87.6(85.4,89.8)	418	77.7(74.2,81.2)	1194	83.8
	*Ever married*	110	12.4(10.2,14.6)	120	22.3(18.8,25.83)	230	16.2
Residence	*Urban*	799	90.2 (88.2, 92.1)	467	86.8(83.9,89.7)	1266	88.9
	*Rural*	87	9.8 (7.9, 11.8)	71	13.2(10.3,16.1)	158	11.1
Education	*High school or below*	228	25.7(22.8,28.6)	141	26.2(22.5,29.9)	369	25.9
	*College/Bachelors*	610	68.8(65.8,71.9)	359	66.7(62.7,70.7)	969	68.0
	*Masters or PhD*	48	5.4(3.9,6.9)	38	7.1(4.9,9.2)	86	6.0
Student	*Yes*	391	44.1(40.9,47.4)	120	22.3(18.8,25.8)	511	43.7
	*No*	495	55.9(52.6,59.2)	164	30.5(26.6,34.4)	659	56.3
Annual income	*<$3000 USD*	250	28.2(25.2,31.2)	120	22.3(18.8,25.8)	370	26.0
	*$3000–6000USD*	256	28.9(25.9,31.9)	164	30.5(26.6,34.4)	420	29.5
	*$6001–10000USD*	218	24.6(21.8,27.4)	158	29.4(25.5,33.2)	376	26.4
	*$10001–15000USD*	107	12.1(9.9,14.2)	64	11.9(9.2,14.6)	171	12.0
	*> $15000USD*	55	6.21(4.6,7.8)	32	6.0(3.9,8.0)	87	6.1
Ever tested for STIs except HIV	*Yes*	318	35.9(32.7,39.1)	138	25.6(22.0,29.4)	456	32.0
	*No*	568	64.1(60.9,67.3)	400	74.4(70.6,78.0)	968	68.0
Ever tested for HIV	*Yes*	490	55.3(52.0,58.6)	213	39.6(35.4,43.7)	703	49.4
	*No*	396	44.7(41.4,48.0)	325	60.4(56.3,64.6)	721	50.6
Self-reported living with HIV ^$^	*Yes*	44	9.0 (6.4, 11.5)	24	11.3 (7.0, 15.5)	68	9.7
	*No*	446	91.0 (88.5, 93.6)	189	88.7 (84.5, 93.0)	635	90.3
Transgender individuals	*No*	851	96.0(94.7,97.3)	512	95.2(93.4,97.0)	1363	95.7
	*Yes*	35	4.0(2.7,5.2)	26	4.8(3.0,6.6)	61	4.3
Currently have a main sexual partner	*Yes*	444	50.1(46.8,53.4)	247	45.9(41.7,50.1)	691	48.5
	*No*	442	49.9(46.6,53.2)	291	54.1(49.9,58.3)	733	51.5
Ever had vaginal or anal sex with women	*Yes*	197	22.2(19.5,25.0)	217	40.3(36.2,44.5)	414	29.1
	*No*	689	77.8(75.0,80.5)	321	59.7(55.5,63.8)	1010	70.9
Had condomless sex with women in the last 3 months	*Yes*	82	9.3(7.3,11.2)	100	18.6(15.3,21.9)	182	12.8
	*No*	804	90.7(88.8,92.7)	438	81.4(78.1,84.7)	1242	87.2
Prefer insertive or receptive anal sex with men	*insertive*	288	32.5(29.4,35.6)	236	43.9(39.7,48.1)	524	36.8
	*receptive*	428	48.3(45.0,51.6)	193	35.9(31.8,39.9)	621	43.6
	*no preference*	170	19.2(16.6,21.8)	109	20.3(16.8,23.7)	279	19.6
Had condomless sex with men during the last sexual act in the last 6 months	*No*	474	53.5(50.2,56.8)	279	51.8(47.6, 56.1)	753	52.9
	*Yes*	196	22.1(19.4,24.9)	102	19.0(15.6,22.3)	298	20.9
	*No anal sex*	216	24.4(21.5, 27.2)	157	29.2(25.3, 33.0)	373	26.2
Drunk alcohol during or prior to sex in the last 3 months	*Yes*	58	6.6(4.9,8.2)	66	12.3(9.5,15.0)	124	8.7
	*No*	828	93.4(91.8,95.1)	472	87.7(85.0,90.5)	1300	91.3
Ever participated in group sex in the last 12 months	*Yes*	82	9.3(7.3,11.3)	59	11.0(8.3,13.6)	141	9.9
	*No*	804	90.7 (88.8,92.7)	479	89.0(86.4,91.7)	1283	90.1
Had sex in exchange for gifts or money in the last 12 months	*Yes*	49	5.5(4.0,7.0)	33	6.1(4.1,8.2)	82	5.8
	*No*	837	94.5(93.0,96.0)	535	93.9(91.8,95.9)	1372	96.3
Found partner through gay app in the last 6 months	*Yes*	558	63.0(59.8, 66.2)	266	49.4(45.2,53.7)	824	57.9
	*No*	328	37.0(33.8,40.2)	272	50.6(46.3,54.8)	600	42.1
Found partner through internet (except gay apps) in the last 6 months	*Yes*	452	51.1(47.8,54.4)	261	48.5(44.3,52.7)	713	50.1
	*No*	433	48.9(45.6, 52.2)	277	51.5(47.2,55.7)	710	49.9
Found partner through in person in the last 6 months	*Yes*	91	10.3(8.3,12.3)	71	13.2(10.3,16.1)	162	11.4
	*No*	795	89.7(87.7,91.7)	467	86.8(83.9,89.7)	1262	88.6
Used recreational drugs in the last 12 months	*Yes*	226	25.5(22.6,28.4)	98	18.2(14.9,21.5)	324	22.8
	*No*	660	74.5(71.6,77.4)	440	81.8(78.5,85.1)	1100	77.2

In total, 62.2% of participants (*n* = 886) reported ever disclosing their sexual orientation to anyone other than their partners. From this subgroup of participants who had disclosed their sexual orientation, 11.1% of participants (*n* = 158/1424) disclosed to their parents, 12.9% (*n* = 184/1424) disclosed to siblings or other family members, 52.2% (*n* = 743/1424) disclosed to friends or classmates, 11.0% (*n* = 157/1424) disclosed to co-workers, and 16.3% (*n* = 232/1424) disclosed to a doctor or other healthcare professional.

Among all the participants, 49.4% (*n* = 703) reported having ever tested for HIV, and 32.0% reported ever testing for other STIs. 9.7% (*n* = 63) of participants who had tested for HIV self-reported living with HIV.

Overall, 29.1% (*n* = 414) of men had ever had vaginal or anal sex with women, and 12.8% (*n* = 182) had condomless sex with women in the last 3 months. 29.2% of men reported no anal sex in the last 6 months and 20.9% of men had condomless sex with men during the last sex episode within the last 6 months. In addition, 8.7% of the participants reported sex while drunk in the last 3 months and 22.8% of the participants reported sex while under the influence of recreational drugs in the last 12 months. In the last 6 months, 57.9% of the participants used gay apps to find a partner, 50.1% found partners through other internet sites, and 11.4% found partners in person. Table 1 shows the full breakdown of socio-demographic information, comparing men who disclosed their sexual orientation and those who did not.

### Factors associated with sexual orientation disclosure

Multivariate modelling demonstrated that compared to non-disclosers, disclosers were more likely to test for HIV or other STIs, with adjusted ORs (aOR) of 2.22 (95% CI: 1.76–2.80) and 1.87 (95% CI: 1.46–2.39), respectively. In addition, when engaging in anal sex, disclosers were more likely to prefer a receptive role, compared to non-disclosers (aOR = 1.64, 95%CI: 1.27–2.10). The likelihood of sexual orientation disclosure was higher among MSM who used gay apps (aOR = 1.56, 95% CI: 1.25–2.95), and among MSM who found their partners through the internet (aOR = 1.25, 95% CI: 1.00–1.56).

Furthermore, compared to non-disclosers, disclosers were less likely to engage in vaginal or anal sex with women (aOR = 0.47, 95%CI: 0.35–0.62). When engaged in vaginal or anal sex with women, disclosers were less likely to have condomless sex (aOR = 0.55, 95% CI: 0.39–0.79). In addition, sexual orientation disclosure was lower among MSM reporting sex while drunk (aOR = 0.55, 95% CI: 0.37–0.81) and sex while using drugs (aOR = 0.66, 95% CI: 0.50–0.87) (Table 2).

**Table 2.** Factors correlated with sexual orientation disclosure among Chinese MSM, 2014 (*N* = 1424).

		Crude Model			Adjusted Model*		
		OR	95% CLs		OR	95% CLs	
Ever tested for any STIs other than HIV	*No*	*Ref*			*Ref*		
	*Yes*	1.62	1.28	2.06	1.87	1.46	2.39
Ever tested for HIV	*No*	*Ref*			*Ref*		
	*Yes*	1.89	1.52	2.35	2.22	1.76	2.80
Self-reported living with HIV ^$^	*Negative*	*Ref*			*Ref*		
	*Positive*	0.78	0.46	1.31	0.81	0.47	1.40
Student	*No*	*Ref*			*Ref*		
	*Yes*	1.37	1.10	1.70	1.09	0.83	1.43
Sexual Orientation	*Bisexual*	*Ref*			*Ref*		
	*Homosexual*	3.34	2.62	4.25	3.25	2.53	4.18
Currently have a main male/female sexual partner	*No*	*Ref*			*Ref*		
	*Yes*	1.18	0.96	1.47	1.27	1.02	1.59
Preferred sexual role during anal sex	*Insertive*	*Ref*			*Ref*		
	*Receptive*	1.82	1.43	2.31	1.64	1.27	2.10
	*Both*	1.28	0.95	1.72	1.20	0.89	1.63
Ever had vaginal or anal sex with women	*No*	*Ref*			*Ref*		
	*Yes*	0.42	0.34	0.53	0.47	0.35	0.62
Engaged in condomless sex with women in the last 3 months	*No*	*Ref*			*Ref*		
	*Yes*	0.45	0.33	0.61	0.55	0.39	0.79
Had condomless sex with men during the last sexual act in the last 6 months	*No*	*Ref*			*Ref*		
	*Yes*	1.13	0.85	1.50	1.14	0.86	1.52
	*No anal sex*	0.81	0.63	1.04	0.80	0.62	1.04
Drunk alcohol during or prior to sex in the last 3 months	*No*	*Ref*			*Ref*		
	*Yes*	0.50	0.35	0.73	0.55	0.37	0.81
Participated in group sex in the last 12 months	*No*	*Ref*			*Ref*		
	*Yes*	0.83	0.58	1.18	0.93	0.65	1.35
Had sex in exchange for gifts or money in the last 12 months	*No*	*Ref*			*Ref*		
	*Yes*	0.90	0.57	1.41	0.85	0.53	1.36
Found partner through gay app in the last 6 months	*No*	*Ref*			*Ref*		
	*Yes*	1.74	1.40	2.16	1.56	1.25	1.95
Found partner through internet in the last 6 months	*No*	*Ref*			*Ref*		
	*Yes*	1.11	0.90	1.38	1.25	1.00	1.56
Found partner through in person in the last 6 months	*No*	*Ref*			*Ref*		
	*Yes*	0.75	0.54	1.05	0.85	0.60	1.20
Used recreational drugs in the last 12 months	*No*	*Ref*			*Ref*		
	*Yes*	0.65	0.50	0.85	0.66	0.50	0.87

* Model adjusted for age (as a continuous variable), residence (urban or rural), education level (high school or below, college or bachelors, masters or PhD) and annual income (less than $3000 USD, $3001–6000 USD, $6001–10,000 USD, $10,001–15,000 USD, or more than $15,000 USD); $ Only limited to participants reported ever tested for HIV, *n* = 703.

### Factors associated with sexual orientation disclosure to healthcare professionals

Our study also evaluated factors correlated with disclosure of sexual orientation to healthcare professionals. Univariate and multivariate models found similar relationships between factors associated with disclosure of sexual orientation to healthcare professionals (Table 3). The likelihood of disclosure to a healthcare professional was greater among MSM who had ever tested for HIV or STIs (aOR = 3.36, 95% CI: 2.50–4.51 for HIVm and aOR = 4.92, 95% CI: 3.47–6.96 for STIs, respectively) and who reported living with HIV (aOR = 1.59, 95% CI: 0.93–2.72). While condomless sex with a woman in the last 3 months was a significant correlate of sexual orientation disclosure to health professionals in univariate analysis, after adjusting for age, residence, income, and education, it was no longer significantly correlated. In both univariate and multivariate logistic regression models, self-reported living with HIV was positively associated with sexual orientation disclosure to health professionals, with a crude OR of 1.61 (95% CI: 0.95–2.73) and an aOR of 1.59 (95% CI: 0.93–2.72).

**Table 3.** Factors correlated with sexual orientation disclosure to healthcare professionals among Chinese MSM, 2014 (*N* = 1424).

		Crude Model			Crude Model*		
		OR	95% CLS		OR	95% CLS	
Ever tested for HIV	*No*	*Ref*			*Ref*		
	*Yes*	3.54	2.65	4.72	3.36	2.50	4.51
Self-reported living with HIV^$^	*No*	*Ref*			*Ref*		
	*Yes*	1.61	0.95	2.73	1.59	0.93	2.72
Ever tested for any STIs other than HIV	*No*	*Ref*			*Ref*		
	*Yes*	5.12	3.65	7.20	4.92	3.47	6.96
Student	*No*	*Ref*			*Ref*		
	*Yes*	0.88	0.66	1.18	1.16	0.81	1.64
Sexual Orientation	*Bisexual*	*Ref*			*Ref*		
	*Homosexual*	1.52	1.08	2.14	1.56	1.10	2.22
Currently have a main male/female sexual partner	*No*	*Ref*			*Ref*		
	*Yes*	1.50	1.13	1.99	1.44	1.08	1.92
Preferred sexual role during anal sex	*Insertive*	*Ref*			*Ref*		
	*Receptive*	0.94	0.68	1.30	1.04	0.75	1.45
	*Both*	1.42	0.97	2.06	1.56	1.06	2.28
Ever had vaginal or anal sex with women	*No*	*Ref*			*Ref*		
	*Yes*	1.09	0.80	1.48	0.90	0.62	1.30
Engaged in condomless sex with women in the last 3 months	*No*	*Ref*			*Ref*		
	*Yes*	1.06	0.70	1.61	0.95	0.60	1.51
Had condomless sex with men during the last sexual act in the last 6 months	*No*	*Ref*			*Ref*		
	*Yes*	1.10	0.77	1.56	1.12	0.78	1.60
Drunk alcohol during or prior to sex in the last 3 months	*No*	*Ref*			*Ref*		
	*Yes*	1.26	0.79	2.02	1.16	0.72	1.89
Participated in group sex in the last 12 months	*No*	*Ref*			*Ref*		
	*Yes*	2.07	1.39	3.10	1.90	1.26	2.86
Had sex in exchange for gifts or money in the last 12 months	*No*	*Ref*			*Ref*		
	*Yes*	1.48	0.86	2.55	1.59	0.92	2.76
Found partner through gay app in the last 6 months	*No*	*Ref*			*Ref*		
	*Yes*	1.16	0.87	1.54	1.18	0.88	1.59
Found partner through internet in the last 6 months	*No*	*Ref*			*Ref*		
	*Yes*	1.38	1.04	1.84	1.33	0.99	1.77
Found partner through in person in the last 6 months	*No*	*Ref*			*Ref*		
	*Yes*	1.43	0.95	2.15	1.26	0.83	1.92
Used recreational drugs in the last 12 months	*No*	*Ref*			*Ref*		
	*Yes*	0.66	0.48	0.90	0.68	0.50	0.94

* Model adjusted for age (Continuous), residence (Urban or rural), education level (High school or below, college or bachelors, or masters or PhD) and annual income (less than $3000 USD, $3001-6000USD, $6001-10,000USD, $10,001-15,000USD, or more than $15,000USD); $ Only limited to participants reported ever tested for HIV, *n* = 703.

## Discussion

Sexual orientation disclosure is closely correlated with increased social support, which could potentially increase self-esteem and psychological adjustment among MSM [20]. This, in turn, promotes linkage and retention to care [21]. Many existing studies focused on sexual orientation disclosure among MSM in high-income settings. This study adds to the current literature by using online recruitment methods to recruit participants from demographically and economically disparate regions throughout China (over 270 cities from 30 provinces), not requiring in-person disclosure of sexual orientation, and providing data on correlations between disclosure and HIV/STI testing. Our findings indicate that sexual orientation disclosure is positively associated with HIV and STI testing, but negatively associated with alcohol or recreational drug use.

We found that only one-sixth of Chinese MSM had ever disclosed their sexual orientation to a doctor or other healthcare professional. This disclosure rate is slightly lower than clinic-based data findings from Beijing [22], and similar data from other low- and middle-income countries is not available [2,23]. In comparison, this rate is much higher in high-income countries, where local environments are more receptive to MSM life. For example, in the United States, 70–90% of MSM had disclosed their sexual orientation to primary care provider [6,11] and in the United Kingdom, this rate is as high as 40% [24]. Low rates of MSM sexual orientation disclosure to health professionals in China may be due inadequate initial assessment, poor retention in care, insufficient social and psychological support, and health professional discrimination [25]. Strategies to improve sexual orientation disclosure to healthcare professionals are needed, especially as the social environment continues to evolve.

Our study showed that disclosure of sexual orientation to a healthcare professional was associated with gay app use. This finding is consistent with the limited literature on gay app use and sexual orientation disclosure [26]. One potential explanation for this phenomenon is that gay apps may increase social support. Gay apps allow MSM to connect with other men with similar backgrounds or experience, potentially providing a source of online social support [27]. The existing literature also suggests that social media use could augment the search for social support in online MSM communities (i.e. gay apps), which in turn promotes disclosure of sexual orientation to others, including healthcare professionals [27].

Our results also showed that disclosure of sexual orientation to healthcare professionals is positively correlated with testing for HIV or other STIs. To the best of our knowledge, this is the first study that explores these associations among MSM in an LMIC setting. This finding is consistent with the results of a study conducted among MSM in the USA [28]. Similarly, we found that living with HIV was positively associated with disclosure of sexual orientation to health professionals. As HIV positive test results are reported to the Center for Disease Control, disclosure of sexual orientation may be part of mandatory case reporting. Conversely, MSM may be driven to test for HIV because of their sexual orientation. While it is not clear whether sexual orientation disclosure promotes HIV testing or if HIV testing promotes sexual orientation disclosure, promoting the two together could be useful [29], especially as rates of both are sub-optimal among MSM in China [30]. While overcoming the sociocultural barriers to promoting HIV testing and disclosure of sexual orientation is challenging in China, use of community campaigns or gay apps to increase social support, and to address these two problems jointly is a promising route [29].

Our study has several limitations. First and foremost, this study was cross-sectional and no causal relationships can be inferred. Second, as an online survey, recruited participants were primarily MSM who were young and well educated [31], potentially excluding older MSM who may be more likely to be married. This older population may be less likely to disclose given a longer exposure to societal stigma. Third, as all collected data (socio-demographic, behaviours, and HIV testing results) were self-reported, social desirability bias may be present. However, we anticipate that this bias to be minimal as the survey was online and no face-to-face meetings were involved in the study. Fourth, as some potential participants did not complete our survey, there may have been a selection bias as non-completers and participants may have had different socio-demographic characteristics and behaviours. However, as the data of the non-completers was excluded from our survey data, we were unable to compare these two groups. Fifth, for the purposes of this study, “healthcare professional” was a general term encompassing several types of care providers, including HIV clinics, hospitals, testing personnel, general medical practitioners, and more. As the role of each of these providers may vary, it is difficult to generalize our findings with any certainty. By considering the varied types of providers as one general category, we may have overlooked important information and the estimated associations may be skewed. Future studies should address this issue with subgroup analyses to provide more information on the topic. Finally, as many potential participants who clicked the survey link withdrew before eligibility screening, another selection bias may have also occurred. Regardless, our study provides preliminary evidence for the significance of promoting disclosure of sexual orientation, especially to healthcare professionals, among Chinese MSM.

## Conclusion

Our study demonstrated that sexual orientation disclosure is correlated with HIV and other STI testing, and lower-risk sexual behaviours. As such, policies facilitating sexual orientation disclosure and testing are recommended. Specifically, policy makers, researchers, and the MSM community should work together to build a more supportive environment to facilitate sexual orientation disclosure. In addition, longitudinal prospective studies targeting how sexual orientation disclosure affects behavioural norms of MSM are needed to provide tailored interventions. Promoting MSM sexual orientation disclosure may be part of a comprehensive HIV intervention tailored to meet the needs of MSM in LMIC settings.

## References

[1] Zhao Y, Ma Y, Chen R, Li F, Qin X, Hu Z. Non-disclosure of sexual orientation to parents associated with sexual risk behaviors among gay and bisexual MSM in China. AIDS Behav. 2016;20(1):193–9. 26174317 10.1007/s10461-015-1135-6

[2] Henry E, Awondo P, Fugon L, Yomb Y, Spire B Coming out of the Nkuta: disclosure of sexual orientation associated with reduced risk behavior among MSM in Cameroon. Arch Sex Behav. 2012;41(3):525–7. 22315042 10.1007/s10508-012-9916-8

[3] Preston DB, D’augelli AR, Kassab CD, Starks MT The relationship of stigma to the sexual risk behavior of rural men who have sex with men. AIDS Educ Prev. 2007;19(3):218–30. 17563276 10.1521/aeap.2007.19.3.218

[4] Oswalt SB, Wyatt TJ Sexual orientation and differences in mental health, stress, and academic performance in a national sample of US college students. J Homosex. 2011;58(9):1255–80. 21957858 10.1080/00918369.2011.605738

[5] Badgett ML Employment and sexual orientation: disclosure and discrimination in the workplace. J Gay Lesbian Soc Serv. 1996;4(4):29–52.

[6] Petroll AE, Mosack KE Physician awareness of sexual orientation and preventive health recommendations to men who have sex with men. Sex Transm Dis. 2011;38(1):63. 20706178 10.1097/OLQ.0b013e3181ebd50f PMC4141481

[7] Juster R-P, Smith NG, Ouellet É, Sindi S, Lupien SJ Sexual orientation and disclosure in relation to psychiatric symptoms, diurnal cortisol, and allostatic load. Psychosom Med. 2013;75(2):103–16. 23362500 10.1097/PSY.0b013e3182826881

[8] MMWR HIV/STD Risks in young men who have sex with men who do not disclose their sexual orientation—six US cities, 1994-2000. MMWR. 2003;52(5):81–5. 12588004

[9] Saewyc EM Research on adolescent sexual orientation: development, health disparities, stigma and resilience. J Res Adolesc: off J Soc Res Adolesc. 2011;21(1):256–72. 10.1111/j.1532-7795.2010.00727.x PMC4835230 27099454

[10] Rothman EF, Sullivan M, Keyes S, Boehmer U Parents’ supportive reactions to sexual orientation disclosure associated with better health: results from a population-based survey of LGB adults in Massachusetts. J Homosex. 2012;59(2):186–200. 22335417 10.1080/00918369.2012.648878 PMC3313451

[11] Durso LE, Meyer IH Patterns and predictors of disclosure of sexual orientation to healthcare providers among lesbians, gay men, and bisexuals. Sexuality Res Soc Policy. 2013;10(1):35–42. 10.1007/s13178-012-0105-2 PMC3582401 23463442

[12] Mason K, Ketende S, Peitzmeier S, Ceesay N, Logie C, Diouf D, et al Stigma, human rights violations, health care access, and disclosure among men who have sex with men in the Gambia. J Hum Rights Pract. 2015;7:139–52.huu026

[13] Neilands TB, Steward WT, Choi K-H Assessment of stigma towards homosexuality in China: a study of men who have sex with men. Arch Sex Behav. 2008;37(5):838–44. 18274889 10.1007/s10508-007-9305-x

[14] Tang W, Best J, Zhang Y, Liu F, Tso LS, Huang S, et al Gay mobile apps and the evolving virtual risk environment: a cross-sectional online survey among men who have sex with men in China. Sex Transm Infect. 2016;92:508–14.sextrans-2015-052469 10.1136/sextrans-2015-052469 PMC5148710 27288414

[15] Zhang L, Chow EP, Jing J, Zhuang X, Li X, He M, et al HIV prevalence in China: integration of surveillance data and a systematic review. Lancet Infect Dis. 2013;13(11):955–63. 24107261 10.1016/S1473-3099(13)70245-7

[16] Miller CJ We can only be healthy if we love ourselves: queer AIDS NGOs, kinship, and alternative families of care in China. AIDS Care. 2016;28(sup4):51–60. 27309725 10.1080/09540121.2016.1195481

[17] Cheng W, Cai Y, Tang W, Zhong F, Meng G, Gu J, et al Providing HIV-related services in China for men who have sex with men. Bull World Health Organ. 2016;94(3):222–7. 26966334 10.2471/BLT.15.156406 PMC4773928

[18] Eysenbach G Improving the quality of web surveys: the checklist for reporting results of internet E-surveys (CHERRIES). J Med Internet Res. 2004;6(3):e34. 15471760 10.2196/jmir.6.3.e34 PMC1550605

[19] Tang W, Han L, Best J, Zhang Y, Mollan K, Kim J, et al Crowdsourcing HIV test promotion videos: a noninferiority randomized controlled trial in China. Clin Infect Dis. 2016;62:1436–1442.ciw171 27129465 10.1093/cid/ciw171 PMC4872295

[20] Rosario M, Schrimshaw EW, Hunter J Disclosure of sexual orientation and subsequent substance use and abuse among lesbian, gay, and bisexual youths: critical role of disclosure reactions. Psychol Addict Behav. 2009;23(1):175–84. 19290704 10.1037/a0014284 PMC2818609

[21] Wohl AR, Galvan FH, Myers HF, Garland W, George S, Witt M, et al Do social support, stress, disclosure and stigma influence retention in HIV care for Latino and African American men who have sex with men and women? AIDS Behav. 2011;15(6):1098–110. 20963630 10.1007/s10461-010-9833-6

[22] Guo Y, Li X, Liu Y, Jiang S, Tu X Disclosure of same-sex behavior by young Chinese migrant men: context and correlates. Psychol Health Med. 2014;19(2):190–200. 23654216 10.1080/13548506.2013.793367

[23] Closson EF, Colby DJ, Nguyen T, Cohen SS, Biello K, Mimiaga MJ The balancing act: exploring stigma, economic need and disclosure among male sex workers in Ho Chi Minh City, Vietnam. Glob Public Health. 2015;10(4):520–31. 25555192 10.1080/17441692.2014.992452 PMC4346426

[24] Metcalfe R, Laird G, Nandwani R Don’t ask, sometimes tell. A survey of men who have sex with men sexual orientation disclosure in general practice. Int J STD AIDS. 2015;26(14):1028–34. 25527656 10.1177/0956462414565404

[25] Ueno K Sexual orientation and psychological distress in adolescence: examining interpersonal stressors and social support processes. Soc Psychol Q. 2005;68(3):258–77.

[26] Bien CH, Best JM, Muessig KE, Wei C, Han L, Tucker JD Gay apps for seeking sex partners in China: implications for MSM sexual health. AIDS Behav. 2015;19(6):941–6. 25572834 10.1007/s10461-014-0994-6 PMC4475493

[27] Gudelunas D There’s an app for that: the uses and gratifications of online social networks for gay men. Sex Cult. 2012;16(4):347–65.

[28] Bernstein KT, Liu K-L, Begier EM, Koblin B, Karpati A, Murrill C Same-sex attraction disclosure to health care providers among New York City men who have sex with men: implications for HIV testing approaches. Arch Intern Med. 2008;168(13):1458–64. 18625927 10.1001/archinte.168.13.1458

[29] Metheny N, Stephenson R Disclosure of sexual orientation and uptake of HIV testing and hepatitis vaccination for rural men who have sex with men. Ann Fam Med. 2016;14(2):155–8. 26951591 10.1370/afm.1907 PMC4781519

[30] Zou H, Hu N, Xin Q, Beck J HIV testing among men who have sex with men in China: a systematic review and meta-analysis. AIDS Behav. 2012;16(7):1717–28. 22677975 10.1007/s10461-012-0225-y

[31] Han L, Candidate B, Bien CH, Wei C, Muessig KE, Yang M, et al HIV self-testing among online MSM in China: implications for expanding HIV testing among key populations. J Acquir Immune Defic Syndr. 2014;67(2):216. 24991972 10.1097/QAI.0000000000000278 PMC4162828

